# Supplementary material for: Genetic Basis Identification of a NLR Gene, TaRGA5-like, That Confers Partial Powdery Mildew Resistance in Wheat SJ106
Source: Int J Mol Sci. 2024 Jun 15;25(12):6603. doi: 10.3390/ijms25126603 (PMC11204014; doi:10.3390/ijms25126603)
Supplement: Supplementary file 1 [file ijms-25-06603-s001.zip › Supplementary figures-revised.pdf]

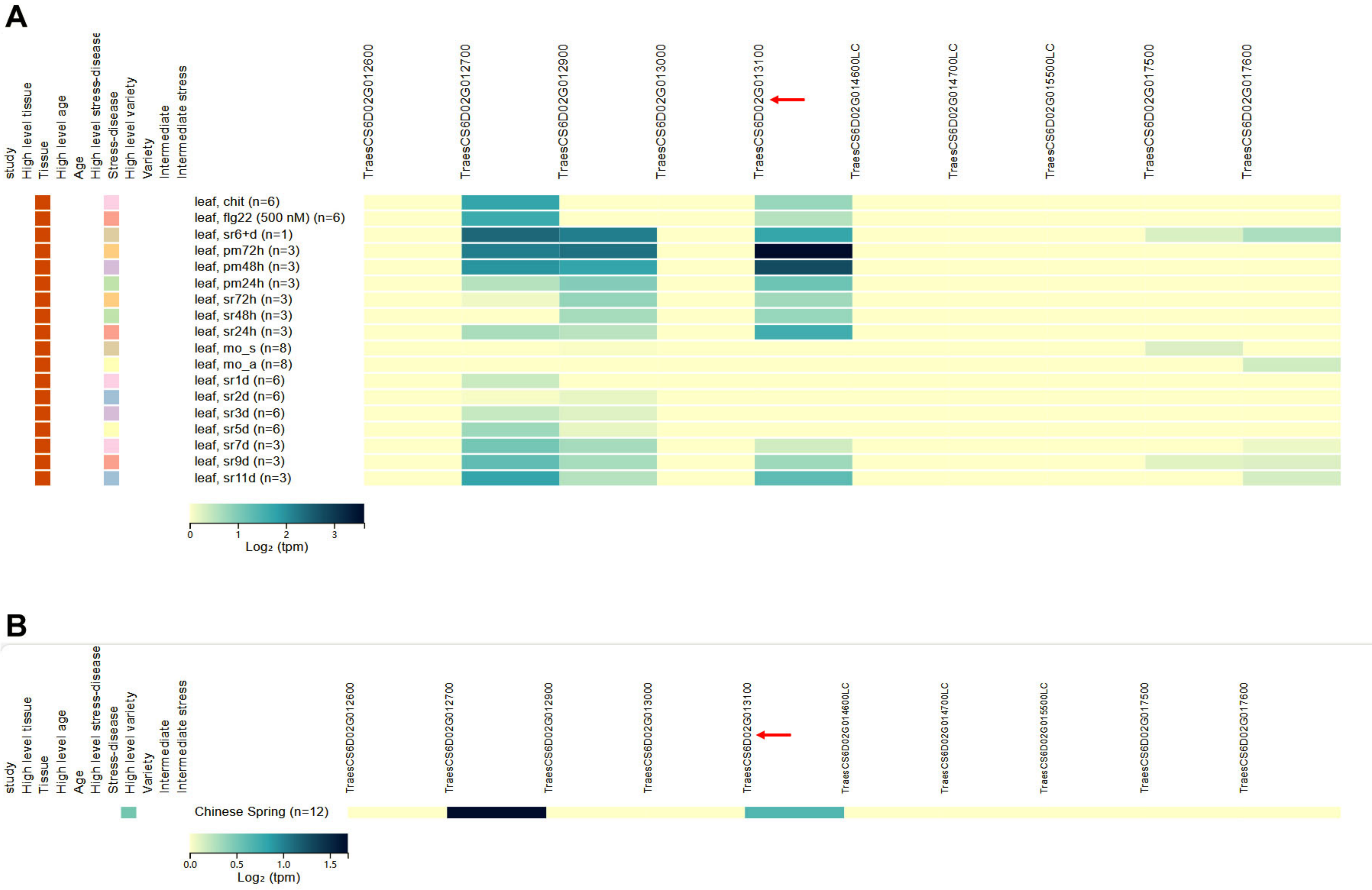

Figure S1: The on-line expression analysis of NLRs annotated in Chinese Spring genome. The red arrow pointed to *TraesCS6D03G0027500*.

```
TraesCS6D03G0027500   MEFATGALGTLLPKLGELLLDEYNLQKGLKKGIKDLMDEL     40
TaRGA5-like           MEFATGALGTLLPKLGELLLDEYNLQKGLKKGIKDLMDEL     40
Consensus             mefatgalgtllpklgellldeynlqkglkkgikdlmdel

TraesCS6D03G0027500   LVIQAVLLKVSNVPLDQLDPQVKIWANDVRELSFAIEDSL     80
TaRGA5-like           LVIQAVLLKVSNVPLDQLDPQVKIWANDVRELSFAIEDSL     80
Consensus             lviqavllkvsnvpldqldpqvkiwandvrelsfaiedsl

TraesCS6D03G0027500   DSFMVRVEGVEPTKPHTFFGFIKKTCKKVTKLKIRREIAN    120
TaRGA5-like           DSFMVRVEGVEPTKPHTFFGFIKKTCKKVTKLKIRREIAN    120
Consensus             dsfmvrvegveptkphtffgfikktckkvtklkirreian

TraesCS6D03G0027500   DIKDVKIQVKEVKERFDRYKDVIGNTNARTEVDPRLLAMY    160
TaRGA5-like           DIKDVKIQVKEVKERFDRYKDVIGNTNARTEVDPRLLAMY    160
Consensus             dikdvkiqvkevkerfdrykdvigntnartevdprllamy

TraesCS6D03G0027500   TKVSDLIGIEMSTDELTERLSKGHDPSGETLKVVSVVGFG    200
TaRGA5-like           TKVSDLIGIEMSTDELTERLSKGHDPSGETLKVVSVVGFG    200
Consensus             tkvsdligiemstdelterlskghdpsgetlkvvsvvgfg

TraesCS6D03G0027500   GLGKTTLAKAVYDKLNKNFDCGVFVPVGQNPDTKKVLRDI    240
TaRGA5-like           GLGKTTLAKAVYDKLNKNFDCGVFVPVGQNPDTKKVLRDI    240
Consensus             glgkttlakavydklnknfdcgvfvpvgqnpdtkkvlrdi

TraesCS6D03G0027500   LLELDRQLYRDASTMDERQLINQLQTFLVGKRYFIVIDDI    280
TaRGA5-like           LLELDRQLYRDASTMDERQLINQLQTFLVGKRYFIVIDDI    280
Consensus             lleldrqlyrdastmderqlinqlqtflvgkryfividdi

TraesCS6D03G0027500   WDTPTWEMINCAFMDSHPESRIIITTRDFDVATKAGGIYR    320
TaRGA5-like           WDTPTWEMINCAFMDSHPESRIIITTRDFDVATKAGGIYR    320
Consensus             wdtptwemincafmdshpesriiittrdfdvatkaggiyr

TraesCS6D03G0027500   MEPLSDDNSKMLFYTRTFGGEGASSDNQPAEVTNKILKKC    360
TaRGA5-like           MEPLSDDNSKMLFYTRTFGGEGASSDNQPAEVTNKILKKC    360
Consensus             meplsddnskmlfytrtfggegassdnqpaevtnkilkkc

TraesCS6D03G0027500   GGVPLAIITIASLLVGKRCEDWSKVYDAIGFGHEDNKVVH    400
TaRGA5-like           GGVPLAIITIASLLVGKRCEDWSKVYDAIGFGHEDNKVVH    400
Consensus             ggvplaiitiasllvgkrcedwskvydaigfghednkvvh

TraesCS6D03G0027500   NTRKILSFSYYDLPSHLKSCLLYLSMFPEDSLIGKRTLIW    440
TaRGA5-like           NTRKILSFSYYDLPSHLKSCLLYLSMFPEDSLIGKRTLIW    440
Consensus             ntrkilsfsyydlpshlkscllylsmfpedsligkrtliw

TraesCS6D03G0027500   RWVCEGFVVDREGIGSFELGEIYFNELVNKSMIRWIDEEN    480
TaRGA5-like           RWVCEGFVVDREGIGSFELGEIYFNELVNKSMIRWIDEEN    480
Consensus             rwvcegfvvdregigsfelgeiyfnelvnksmirwideen

TraesCS6D03G0027500   YGQGGCRVHDMVLDLIRTISNDINFVTVHDMEQHGTSLRG    520
TaRGA5-like           YGQGGCRVHDMVLDLIRTISNDINFVTVHDMEQHGTSLRG    520
Consensus             ygqggcrvhdmvldlirtisndinfvtvhdmeqhgtslrg

TraesCS6D03G0027500   QQTNRVHRLALHGRSVEHNSSIAMEHLRSFNVVGCSANSM    560
TaRGA5-like           QQTNRVHRLALHGRSVEHNSSIAMEHLRSFNVVGCSANSM    560
Consensus             qqtnrvhrlalhgrsvehnssiamehlrsfnvvgcsansm

TraesCS6D03G0027500   PLLLSFKVLRVLVIEDCVFSVGSSLEHLGKLVQLRYLGLV    600
TaRGA5-like           PLLLSFKVLRVLVIEDCVFSVGSSLEHLGKLVQLRYLGLV    600
Consensus             plllsfkvlrvlviedcvfsvgsslehlgklvqlrylglv

TraesCS6D03G0027500   KTAVKIPEGIGHDLKFLEILDVRGGLISELPPSVGELMNL    640
TaRGA5-like           KTAVKIPEGIGHDLKFLEILDVRGGLISELPPSVGELMNL    640
Consensus             ktavkipegighdlkfleildvrgglisel ppsvgelmnl

TraesCS6D03G0027500   RCLWADKGTVMKGEIGKLTCLEELELYSVEKCPNFCTEVG    680
TaRGA5-like           RCLWADKGTVMKGEIGKLTCLEELELYSVEKCPNFCTEVG    680
Consensus             rclwadkgtvmkgeigkltcleelelysvekcpnfctevg

TraesCS6D03G0027500   KLTTLRVLKIYFAEIEESAGKALMESLCNLHNIHSLTVLD    720
TaRGA5-like           KLTTLRVLKIYFAEIEESAGKALMESLCNLHNIHSLTVLD    720
Consensus             klttlrvlkiyfaeieesagkalmeslcnlhnihsltvld

TraesCS6D03G0027500   DADDGKYSIVLNHSLEDLACTKLHELALLSIVIPRVPSWI    760
TaRGA5-like           DADDGKYSIVLNHSLEDLACTKLHELALLSIVIPRVPSWI    760
Consensus             daddgkysivlnhsledlactklhelallsiviprvpswi

TraesCS6D03G0027500   NHLSVPLLSHLGLHVAAVEVGDVQTIGRLPSLLVLLLWSK    800
TaRGA5-like           NHLSAPLLNRLWLHVDAVEVRDVQTIGRLPSLLVLGLWSN    800
Consensus             nhls pll  l lhv avev dvqtigrlpsllvl lws

TraesCS6D03G0027500   DEKNISYTFGSNEFHKLRVLWTKKIEIAVGEGALPMLEVL    840
TaRGA5-like           EENNVSYTFGTNEFHKLRCLYTKKIEIAVGEGALPMLEWL    840
Consensus              e n sytfg nefhklr l tkkieiavgegalpmle l

TraesCS6D03G0027500   EYRASAERKDAASLVPWRRNSCPLLKFVGCFLDCTNSSYR    880
TaRGA5-like           RYTASAERKDAASLVPWRRNSCPLLKFVGCFLDCTNSSYR    880
Consensus              y asaerkdaaslvpwrrnscpllkfvgcfldctnssyr

TraesCS6D03G0027500   EVKEAKQALRQASGTRPNAVYLDLYIEEENYDVEAGKFID    920
TaRGA5-like           EVKEAKQALRQASGTRPNAVYLDLYIEEENYDVEAGKFID    920
Consensus             evkeakqalrqasgtrpnavyldlyieeenydveagkfid

TraesCS6D03G0027500   NLEWTLRGLDRPEDVGRAAAHQEERTRRMIRSLERRLRDA    960
TaRGA5-like           NLEWTLRGLDRPEDVGRAAAHQEERTRRMIRSLERRLRDA    960
Consensus             nlewtlrgldrpedvgraaahqeertrrmirslerrlrda

TraesCS6D03G0027500   AEPRVGRYGQQEIRGLVAKFKRWLHDHAGTDQDEAGKSDD   1000
TaRGA5-like           AEPRVGRYGQQEIRGLVAKFKRWLHDHAGTDQDEAGKSDD   1000
Consensus             aeprvgrygqqeirglvakfkrwlhdhagtdqdeagksdd

TraesCS6D03G0027500   DEDYYYGDDDDGTDQDDQDDDDDYGTDQAEVDDCCEAESTC   1040
TaRGA5-like           DEDYYYGDDDDGTDQDDQDDDDDYGTDQAEVDDCCEAESTC   1040
Consensus             dedyyygddddgtdqddqdddddygtdqaevddcceaestc

TraesCS6D03G0027500   SDDC                                       1044
TaRGA5-like           SDDC                                       1044
Consensus             sddc
```

Figure S2: Amino acids alignment between SJ106 and Chinese Spring

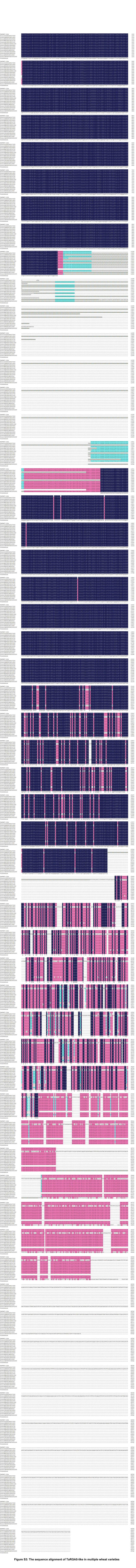

Figure S3: The sequence alignment of TaRGAS-like in multiple wheat varieties

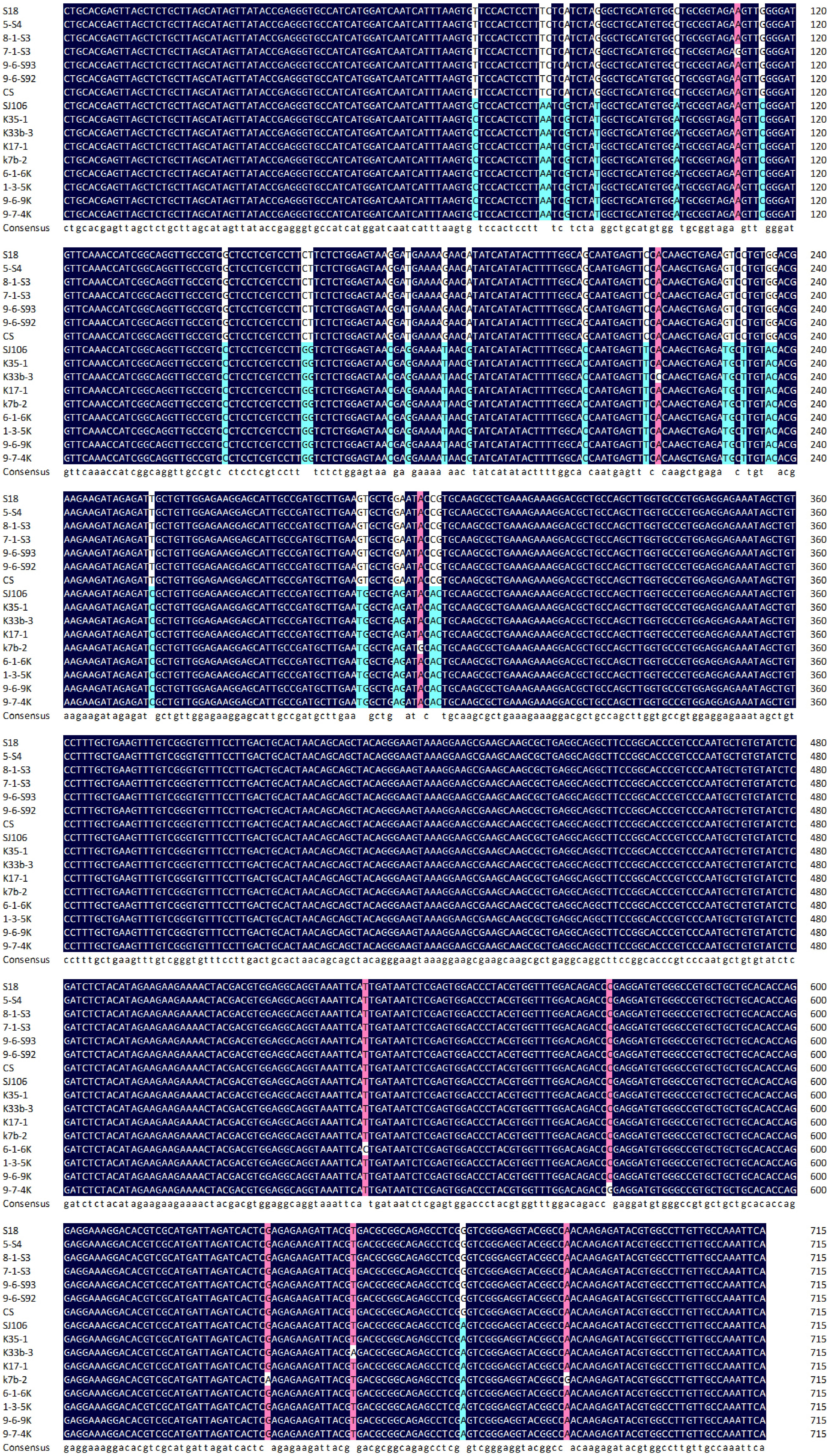

Figure S4: The LRR region alignment in single plants

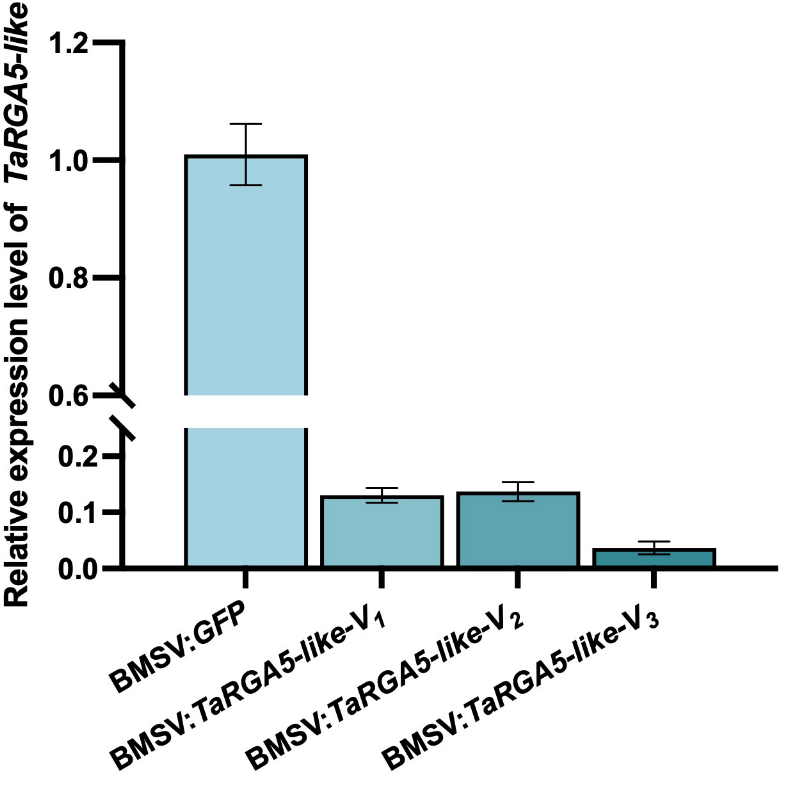

Figure S5 Analysis of TaRGA5-like silencing efficiency. BSMV:*GFP*: SJ106 infected with BSMV:*GFP*, BSMV:*TaRGA5-like-V1*: SJ106 infected with BSMV:*TaRGA5-like-V1*, BSMV:*TaRGA5-like-V2*: SJ106 infected with BSMV:*TaRGA5-like-V2*, BSMV:*TaRGA5-like-V3*: SJ106 infected with BSMV:*TaRGA5-like-V3*

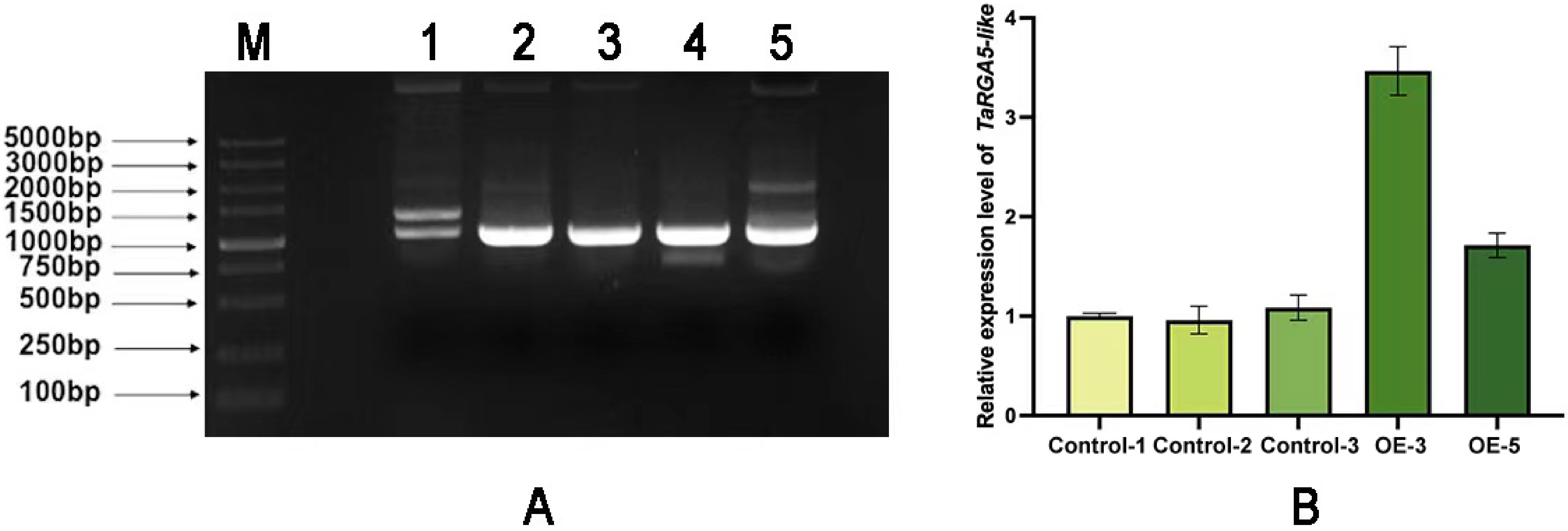

Figure S6: Identification of transgenic wheat. A, genomic PCR identification of TaRGA5-like. M:DL 5000 Marker； line 1: control: Jinqiang5;； line 2-5: OE-3, 5: transgeninc Jinqiang5 wheat containing the recombinant vector pTCK303:TaRGA5-like. B, relative expression levels of TaRGA5-like in over-expression wheat plants.
