# Supplementary material for: Genetic Basis Identification of a NLR Gene, TaRGA5-like, That Confers Partial Powdery Mildew Resistance in Wheat SJ106
Source: Int J Mol Sci. 2024 Jun 15;25(12):6603. doi: 10.3390/ijms25126603 (PMC11204014; doi:10.3390/ijms25126603)
Supplement: Supplementary file 1 [file ijms-25-06603-s001.zip › Supplementary legends.pdf]

Table S1: Primer sequence in this study;
Table S2: Genetic analysis of resistance to *Bgt E09* in Chinese Spring /SJ106;
Table S3: The annotated information of NLRs in multiple wheat genomes;
Table S4: Statistics calculations of VIGS and OE;
Figure S1: The on-line expression analysis of NLRs annotated in Chinese Spring genome. The red arrow pointed to *TraesCS6D03G0027500*;
Figure S2: Amino acids alignment between SJ106 and Chinese Spring;
Figure S3: The sequence alignment of TaRGA5-like in multiple wheat varieties;
Figure S4: The LRR region alignment in single plants;
Figure S5: Analysis of TaRGA5-like silencing efficiency. BSMV:GFP: SJ106 infected with BSMV:*GFP*, BSMV:*TaRGA5-like-V1*: SJ106 infected with BSMV:*TaRGA5-like-V1*, BSMV:*TaRGA5-like-V2*: SJ106 infected with BSMV:*TaRGA5-like-V2*, BSMV:*TaRGA5-like-V3*: SJ106 infected with BSMV:TaRGA5-like-V3;
Figure S6: Identification of transgenic wheat. A, genomic PCR identification of TaRGA5-like. M:DL 5000 Marker；line 1: negative controls；line 2-5: pTCK303-TaRGA5-like transgenic wheat. B, relative expression levels of TaRGA5-like in over-expression wheat plants.
