# Supplementary material for: Genetic Basis Identification of a NLR Gene, TaRGA5-like, That Confers Partial Powdery Mildew Resistance in Wheat SJ106
Source: Int J Mol Sci. 2024 Jun 15;25(12):6603. doi: 10.3390/ijms25126603 (PMC11204014; doi:10.3390/ijms25126603)
Supplement: Supplementary file 1 [file ijms-25-06603-s001.zip › Table S1.pdf]

Table S1

Primer sequence in this study

Primer name	Sequence（5'→3'）	usage
TaRGA5-like-F	ATGGAGTTTGCCACGGGG	Amplication of full length
TaRGA5-like-R	TTAATTACAATCGTCACTGCAAGTGG	
TaRGA5-like-qRT-F	CCCTCCTCGTCCTTGGTCTC	Expression level analysis
TaRGA5-like-qRT-R	ATCGGCAATGCTCCTTCTCC	
TaRGA5-like-OE-F	TCTAGAGGATCCCCGGGTACCATGGAGTTTGCCACGGGG	Construction of over-expression vector
TaRGA5-like-OE-R	TTCGAGCTCTCTAGAACTAGTTTAATTACAATCGTCACTGCAAGTGG	
TaRGA5-like-V_1-F	CAAACATTTTTTTTTTTTTTTAGCTAGCTCCAAGTTAAAGAGGTGA	Construction of VIGS vector
TaRGA5-like-V_1-R	GATTCTTCTTCCGTTGCTAGCCGAATCCAACAACAGAG	
TaRGA5-like-V_2-F	CAAACATTTTTTTTTTTTTTTAGCTAGCATGATCTACCTTCCCATTT	
TaRGA5-like-V_2-R	GATTCTTCTTCCGTTGCTAGCGGACACGACAACCACC	
TaRGA5-like-V_3-F	CAAACATTTTTTTTTTTTTTTAGCTAGCGCACCAATGAGTTTCACA	
TaRGA5-like-V_3-R	GATTCTTCTTCCGTTGCTAGCTGCTTCGCTTCCTTTAC	
SL-*TaRGA5-like*-F	ACGGGGGACTCTTGACCATGGTAATGGAGTTTGCCACGG	Construction of subcellular location vector
SL-*TaRGA5-like*-R	AAGTTCTTCTCCTTTACTAGTATTACAATCGTCACTGCAAGTGGAC	
Ubi-F	CGGTCGTTCATTCGTTCTA	Identification of OE plants
TaRGA5-like-R（Ubi）	GCTTGTCATACACGGCTTTA	
Tactin-qRT-F	TACTCCCTCACAACAACCG	Internal gene of qRT-PCR
Tactin-qRT-R	AGAACCTCCACTGAGAACAA	
