# Supplementary material for: Genetic Basis Identification of a NLR Gene, TaRGA5-like, That Confers Partial Powdery Mildew Resistance in Wheat SJ106
Source: Int J Mol Sci. 2024 Jun 15;25(12):6603. doi: 10.3390/ijms25126603 (PMC11204014; doi:10.3390/ijms25126603)
Supplement: Supplementary file 1 [file ijms-25-06603-s001.zip › Table S2.pdf]

Table S2 Genetic analysis of resistance to *Bgt E09* in Chinese Spring /SJ106

Parent and hybrid progeny	Passing on from generation generation	Individuals or family coefficient	Plants observed			Actual ratio	Expected ratio	χ^2	*P*
			HR	Seg	HS				
China Spring	P_S	20			40				
SJ106	P_R	20	40						
$P_R \times P_S$	F_1	10	20						
	F_2	285	218		67	3.25:1	3:1	0.338	0.561
	$F_{2:3}$	630	152	313	165	1:2.06:1.09	1:2:1	0.562	0.755

HR: homozygous resistance; Seg: segregant; HS: homozygous susceptible.
