# Supplementary figures and images for: Genetic Basis Identification of a NLR Gene, TaRGA5-like, That Confers Partial Powdery Mildew Resistance in Wheat SJ106
Source: Int J Mol Sci. 2024 Jun 15;25(12):6603. doi: 10.3390/ijms25126603 (PMC11204014; doi:10.3390/ijms25126603)

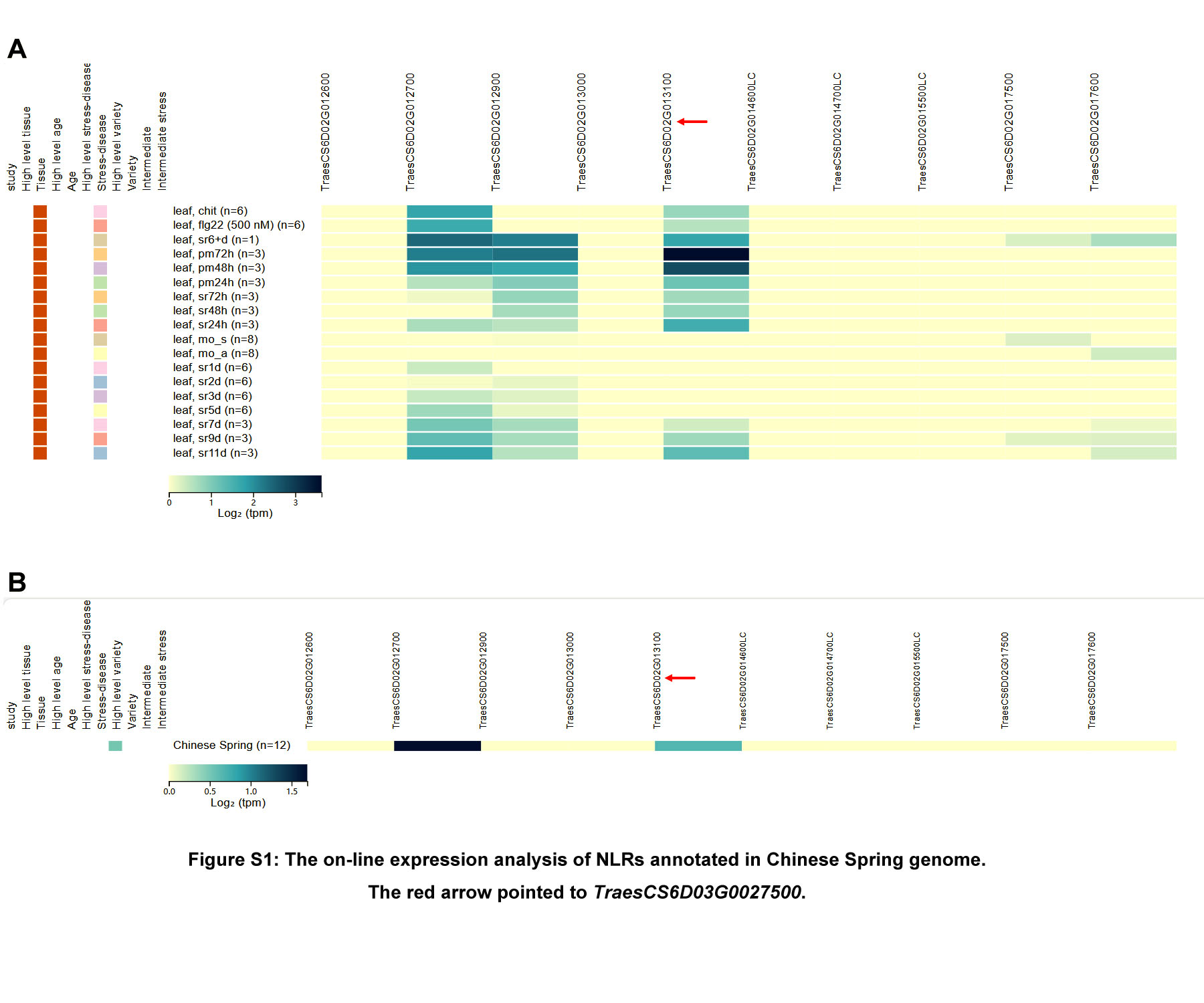

Supplement: Supplementary file 1 [file ijms-25-06603-s001.zip › Fig. S1.jpg]

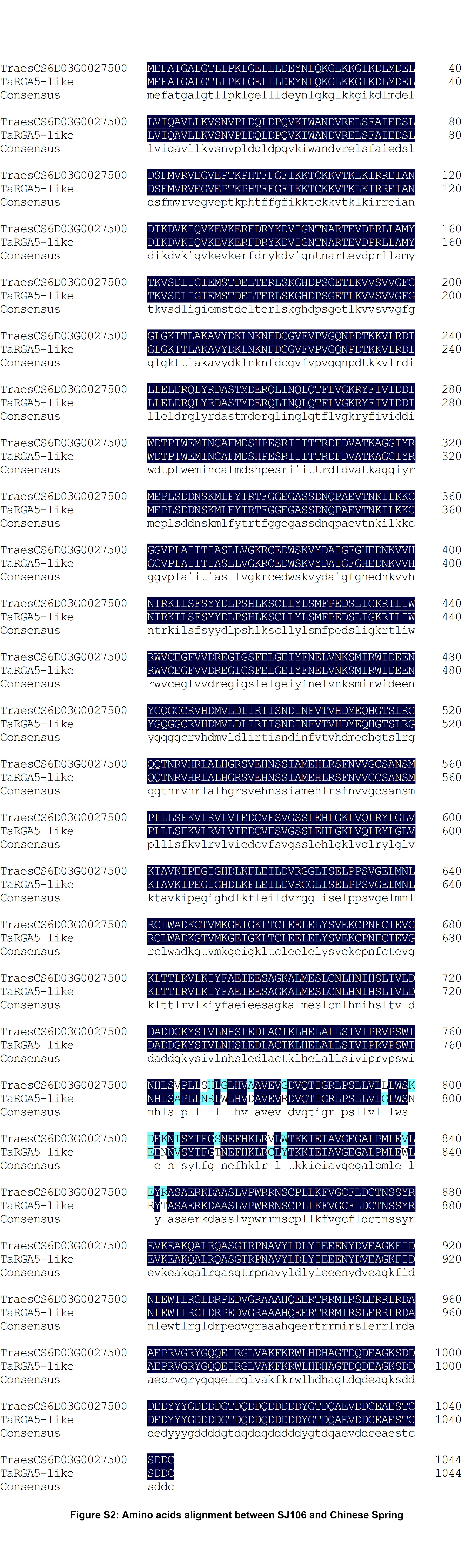

Supplement: Supplementary file 1 [file ijms-25-06603-s001.zip › Fig. S2.jpg]

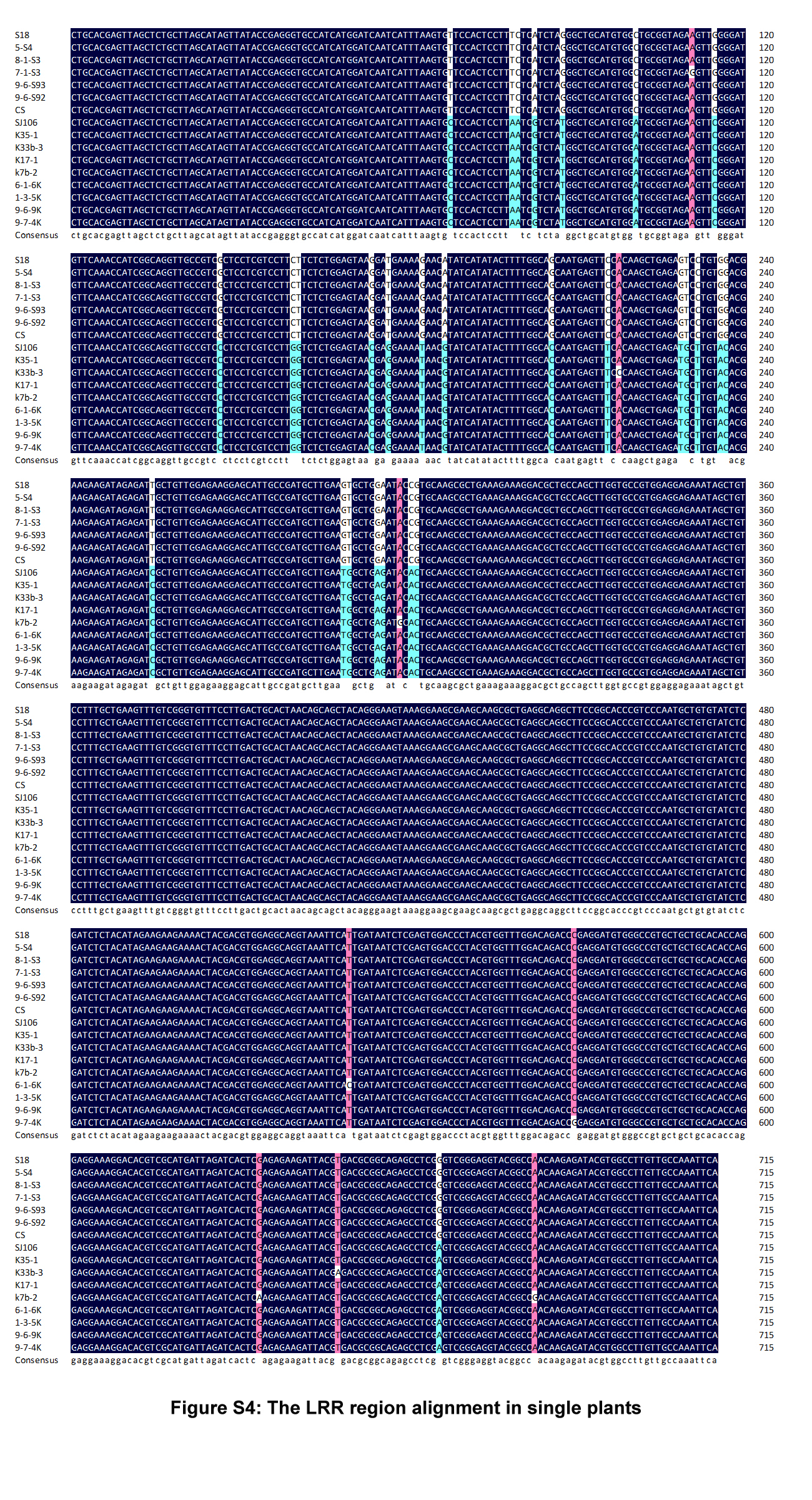

Supplement: Supplementary file 1 [file ijms-25-06603-s001.zip › Fig. S4.jpg]

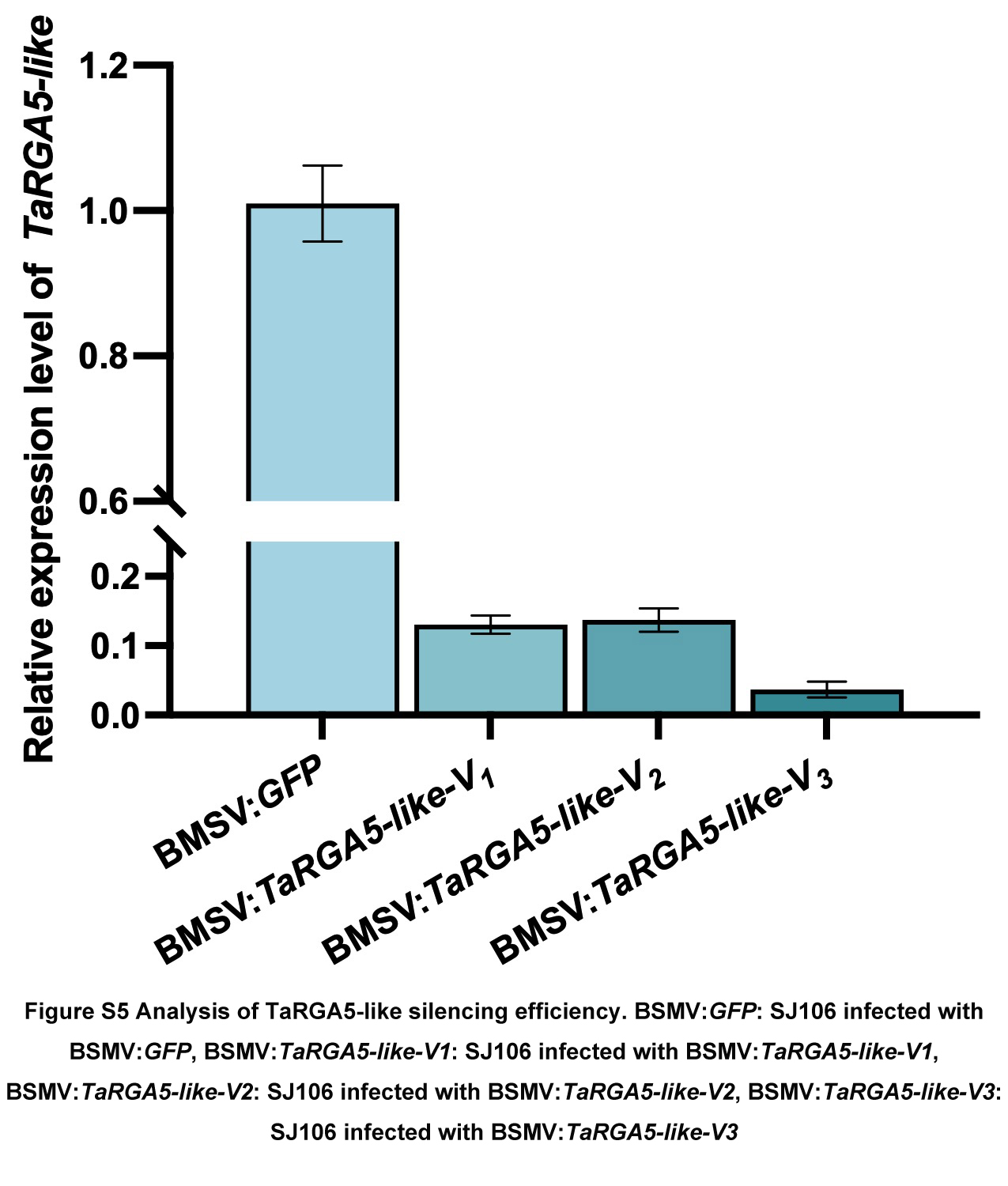

Supplement: Supplementary file 1 [file ijms-25-06603-s001.zip › Fig. S5.jpg]

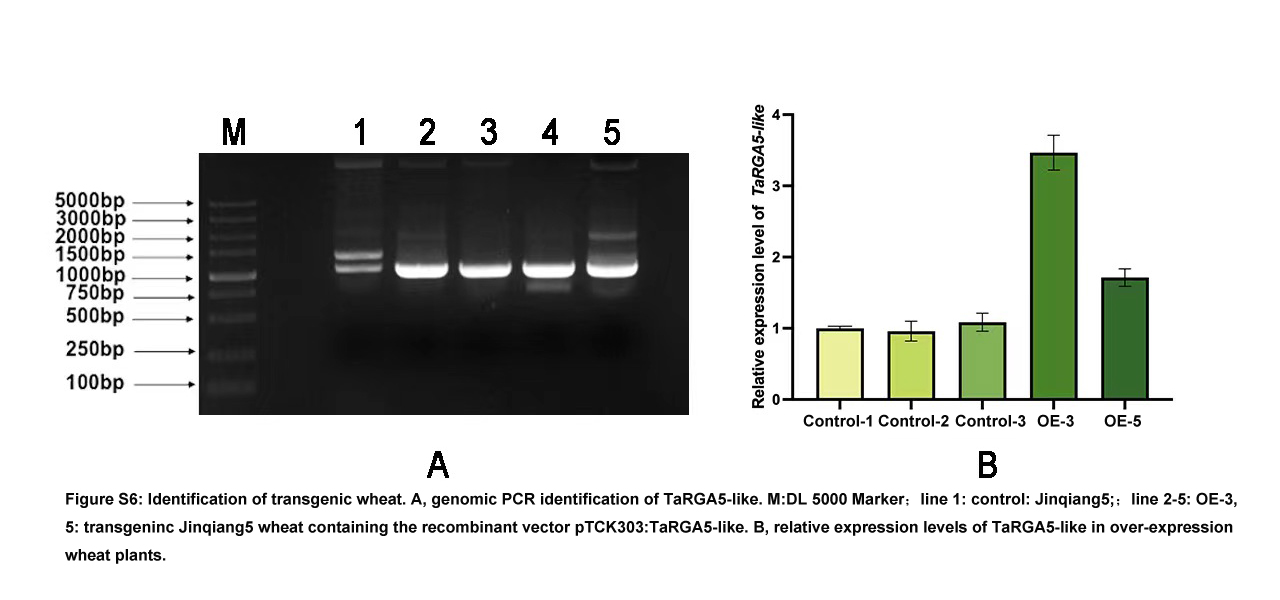

Supplement: Supplementary file 1 [file ijms-25-06603-s001.zip › Fig. S6.jpg]
